# Enhanced Recovery After Surgery (ERAS) in Saudi Arabian Surgical Practice: A Comprehensive Analysis of Surgical Outcomes, Patient Satisfaction, and Cost-Effectiveness

**DOI:** 10.7759/cureus.49448

**Published:** 2023-11-26

**Authors:** Abdulsalam Aleid, Eman M Alyaseen, Razan S Alfurayji, Bader S Alanazi, Fatimah A Alquraish, Abbas Al Mutair, Mohammed Alessa, Loai Albinsaad

**Affiliations:** 1 Department of Surgery, King Faisal University, Hofuf, SAU; 2 College of Medicine and Medical Science, Arabian Gulf University, Manama, BHR; 3 College of Medicine, Qassim University, Qassim, SAU; 4 College of Medicine, Imam Abdulrahman Bin Faisal University, Dammam, SAU; 5 Research Center, Almoosa Specialist Hospital, Al Ahsa, SAU; 6 Department of Surgery, College of Medicine, King Faisal University, Hofuf, SAU

**Keywords:** cost-effectiveness, protocol, complication, perioperative, saudi arabia, impact, enhanced recovery after surgery

## Abstract

Introduction

Surgical procedures present substantial healthcare costs, patient discomfort, and potential adverse outcomes. In response, enhanced recovery after surgery (ERAS) protocols have emerged as comprehensive, evidence-based preoperative care pathways designed to optimize preoperative, intra-operative, and postoperative management. These protocols incorporate various interventions, such as preoperative education, nutritional optimization, minimally invasive techniques, multimodal pain management, early mobilization, and patient engagement. Despite their global success and growing popularity, the adoption and influence of ERAS protocols in Saudi Arabia have not been extensively explored. This study aims to assess the ERAS effects on surgical outcomes and evaluate its relationship with patient satisfaction, considering factors such as cost-effectiveness and compliance in the Saudi context.

Methods

This cross-sectional study encompassed data collection from 1,452 patients who underwent surgical procedures such as bariatric surgery and cholecystectomy, employing systematic random sampling across multiple healthcare facilities in Saudi Arabia. Data were gathered through structured questionnaires, medical records, and cost-effectiveness analysis within the period spanning from January to August 2023. The relationship between ERAS protocol implementation, surgical outcomes, patient satisfaction, and cost-effectiveness was analyzed using statistical tests, including correlation, regression analysis, and chi-square tests. A statistical significance threshold was set at p < 0.05, and Statistical Product and Service Solutions (SPSS, version 28.0) (IBM SPSS Statistics for Windows, Armonk, NY) was used for data analysis.

Results

Among the 1,452 respondents, 1,152 (79.3%) reported the implementation of ERAS protocols during their surgical procedures. Those receiving ERAS protocols exhibited significantly lower rates of surgical complications, readmissions, and reduced dependency on pain medication (p < 0.001). Additionally, participants subjected to ERAS protocols reported significantly higher satisfaction levels based on the mean satisfaction scale score, with a p-value of less than 0.001.

Conclusion

The results highlight substantial improvements associated with the implementation of ERAS protocols, particularly in terms of reduced surgical site infections, shortened hospitalization periods, and decreased pain management-related complications. Moreover, ERAS protocol implementation demonstrated enhanced surgical outcomes, increased postoperative satisfaction, and overall improved recovery experiences. These findings underscore the potential benefits of integrating ERAS protocols into the surgical practices of Saudi Arabia. This research contributes to a better understanding of the advantages offered by ERAS protocols and their potential for enhancing healthcare delivery in the region.

## Introduction

Enhanced recovery after surgery (ERAS) protocols represent a paradigm shift in the management of patients undergoing major surgical procedures, encompassing a comprehensive, evidence-based approach that spans the preoperative, intraoperative, and postoperative phases to enhance recovery and optimize outcomes [1]. These protocols are meticulously designed and implemented by multidisciplinary teams comprising surgeons, anesthetists, nurses or physician assistants, and healthcare professionals specializing in surgical patient care [2].

This transformative approach reevaluates conventional practices, replacing them with evidence-based best practices as needed and addressing every facet of a patient's surgical journey, thereby delivering a structured framework for care management, preoperative planning and preparation, dietary optimization, physical fitness enhancement, and stress reduction, thereby culminating in reduced surgical trauma and improved patient outcomes, ultimately minimizing hospital stays [2].

Initially conceived by Henrik Kehlet in the 1990s, ERAS was first introduced in the context of colorectal surgery [3]. Kehlet et al. pioneered a novel approach aimed at minimizing surgical trauma and physiological stress, resulting in enhanced patient outcomes and reduced lengths of stay following colorectal surgeries [4]. Subsequently, ERAS protocols have witnessed widespread adoption, extending their influence to encompass diverse surgical disciplines, including general surgery [5], urology [6], and gynecology [7].

These protocols have matured into evidence-based guidelines, meticulously curated and disseminated by the International ERAS® Society, a global charitable professional association committed to the development, promotion, and implementation of ERAS programs [8-10]. Extensive research underscores the merits of ERAS adoption, demonstrating reductions in postoperative complications, lengths of stay, pain, and overall costs, accompanied by heightened satisfaction among both patients and healthcare staff [8-10]. Moreover, emerging evidence suggests that ERAS implementation may be associated with improved long-term survival [11,12].

However, despite the demonstrated benefits, the global adoption of ERAS practices remains uneven. Remarkably, the adoption and effectiveness of ERAS protocols in Saudi Arabia remain relatively unexplored. Thus, this cross-sectional study aims to fill this critical knowledge gap by investigating the impact of ERAS protocols on cost-effectiveness, surgical outcomes, and patient satisfaction within the Saudi Arabian healthcare context.

The insights gleaned from this research hold profound significance for healthcare practitioners, policymakers, and stakeholders, contributing to the body of knowledge surrounding ERAS implementation and shaping future healthcare policy and practice. Against this backdrop, this study formulates the following central hypothesis: in Saudi Arabia, where data on ERAS protocol adoption and expansion among healthcare institutions and stakeholders are scarce, the successful implementation of ERAS is imperative for healthcare administrators and professionals to make informed decisions aimed at enhancing the quality of care, shortening hospital stays, and improving the patient experience while concurrently achieving cost efficiencies.

It is envisioned that this study serves as the inaugural installment in a series of investigations aimed at elucidating ERAS practices within the Saudi Arabian healthcare landscape. The research objectives of this study are twofold: first, to assess the impact of ERAS protocols on surgical outcomes within the Saudi Arabian context; second, to evaluate the relationship between ERAS implementation and patient satisfaction while also considering the cost-effectiveness and compliance to ERAS protocols. In addition to these primary objectives, secondary objectives encompass an examination of the association between ERAS implementation and reduced hospital mortality rates, higher rates of home discharge, and a comparative analysis of patient satisfaction between ERAS implementation and conventional approaches [1,3-12].

This comprehensive study endeavors to unveil the uncharted territory of ERAS protocols in Saudi Arabia, setting the stage for a deeper understanding of their implementation, impact, and implications in the context of the nation's healthcare landscape. Ultimately, the findings of this research have the potential to catalyze positive transformations in the Saudi Arabian healthcare system, offering insights that contribute to better patient care, reduced healthcare costs, and enhanced patient experiences.

## Materials and methods

Study area, setting, and time

This cross-sectional study was conducted in Saudi Arabia, encompassing the collection of data from a diverse sample of surgical patients across multiple healthcare facilities within the country. The investigation took place between January 2023 and August 2023, during which a structured questionnaire, medical records, and cost-effectiveness analysis were administered to surgical patients across various healthcare facilities in Saudi Arabia.

Study population, sample size, and sampling technique

The study population included a single group comprising all Saudi and non-Saudi patients with a history of surgical procedures (n=1,452). To ensure adequate representation and statistical power, a minimum sample size equivalent to 1,016 (70%) of the estimated number of patients who underwent surgical procedures was determined. The sampling technique employed was convenience sampling, whereby participants were selected based on their availability and willingness to partake in the study.

Inclusion and exclusion criteria

Inclusion criteria comprised individuals who had undergone surgical operations in Saudi hospitals or healthcare centers and had attained the age of eighteen or older, while exclusion criteria encompassed patients who did not meet the inclusion criteria. Additionally, individuals who declined to participate or could not provide informed consent were excluded from the study.

Data collection tools

The primary data collection tool for this study was a structured questionnaire designed to assess the implementation and impact of ERAS protocols, surgical outcomes, patient satisfaction, and cost-effectiveness. This questionnaire was crafted based on existing literature and tailored to the specific context of ERAS protocols in Saudi Arabia.

Study variables

Independent variables included demographic data such as age, gender, occupation, and medical history. Dependent variables encompassed various aspects related to the implementation of ERAS, patient satisfaction, patient experience, cost-effectiveness, and access to care.

Ethical considerations

Ethical considerations were meticulously integrated into the study. Ethical approval was secured from the King Faisal University Research Ethics Committee: KFU-REC-2023-SEP-ETHICS1,144. Informed consent was diligently obtained from all participants, ensuring their voluntary involvement and confidentiality. Participants were comprehensively informed by a written document about the study's purpose, procedures, and their unequivocal rights to withdraw at any time without incurring any consequences. To mitigate conflicts of interest, measures were taken to guarantee the independence and impartiality of the research team.

Statistical analyses

Descriptive statistics were employed to summarize demographic characteristics, general information, and patient satisfaction. Bivariate associations were explored using chi-square tests to investigate the relationships between categorical variables, including ERAS protocol implementation and surgical outcomes. Mean satisfaction scores were compared between participants with and without ERAS implementation using Student's t-test. The internal consistency of Likert scale sections was evaluated using Cronbach's alpha. Statistical significance was established at p < 0.05. Data analysis was conducted utilizing Statistical Product and Service Solutions (SPSS, version 28.0) (IBM SPSS Statistics for Windows, Armonk, NY). The findings from the statistical analysis offer valuable insights into the associations between ERAS implementation, surgical outcomes, and patient satisfaction, thereby contributing to a more comprehensive understanding of the impact of ERAS protocols on surgical care and patient experiences within the Saudi Arabian context.

## Results

The demographic characteristics of the 1,452 participants are summarized in Table 1. The majority of participants fell within the age groups of 18-24 years 444 (30.6%), 25-34 years 228 (15.7%), and 35-44 years 180 (12.4%). The gender distribution was skewed towards females 816 (56.2%). The majority held a bachelor’s degree 1,032 (71.1%) and were employed full-time 612 (42.1%). Most participants resided in the middle province 996 (68.6%) and identified with an urban geographic location of 1,380 (95.0%).

**Table 1:** Demographic characteristics Age groups are presented in years. Employment status categories include full-time, part-time, retired, student, unemployed, and others.

		Count	N %
Age	18-24	444	30.6%
	25-34	228	15.7%
	35-44	180	12.4%
	44-54	408	28.1%
	55-64	132	9.1%
	above 65	12	0.8%
	under 18	48	3.3%
Gender	Female	816	56.2%
	Male	636	43.8%
Education level	Bachelor's Degree	1032	71.1%
	Doctorate or higher	24	1.7%
	Diploma	120	8.3%
	High school or less	276	19.0%
Employment Status	Employed full-time	612	42.1%
	Employed part-time	36	2.5%
	Other	96	6.6%
	Retired	96	6.6%
	Student	456	31.4%
	Unemployed	156	10.7%
City of residence	Eastern Province	372	25.6%
	Middle Province	996	68.6%
	South Province	12	0.8%
	Western Province	72	5.0%
Geographic location	Rural	48	3.3%
	Suburban	24	1.7%
	Urban	1380	95.0%

Table 2 shows that a significant proportion of participants rated their overall surgical experience as "Excellent" (900, 62.0%), and the majority received preoperative education about their surgical procedure and recovery (1,056, 72.7%). Regarding satisfaction with information provided, 480 (33.1%) reported being "Satisfied," while 480 (33.1%) were "Very satisfied." Most participants did not experience complications following surgery 1,200 (82.6%), and 1,392 (95.9%) reported receiving adequate pain management. The majority 564 (38.8%) were "Satisfied" with the overall surgical outcome. 

**Table 2:** General information and patient satisfaction Satisfaction scale ranges from 1 (Very dissatisfied) to 5 (Very satisfied).

		Count	N %
How would you rate your overall surgical experience	Excellent	900	62.0%
	Fair	96	6.6%
	Good	444	30.6%
	Poor	12	0.8%
Did you receive preoperative education regarding your surgical procedure and recovery?	No	396	27.3%
	Yes	1056	72.7%
How satisfied are you with the information provided about your surgery, including potential risks and benefits?	Dissatisfied	84	5.8%
	Neutral	276	19.0%
	Satisfied	480	33.1%
	Satisfied	108	7.4%
	Very dissatisfied	24	1.7%
	Very satisfied	480	33.1%
Did you experience any complications following your surgery?	No	1200	82.6%
	Yes	252	17.4%
If you experienced complications, please specify the nature of the complications	Infection	360	24.8%
	Bleeding	48	3.3%
	Wound healing issues	24	1.7%
	Organ dysfunction	24	1.7%
How long was your hospital stay following the surgery?	1-2 days	552	38.0%
	3-5 days	312	21.5%
	Less than 24 hours	372	25.6%
	More than 5 days	216	14.9%
Did you receive adequate pain management during your hospital stay?	No	60	4.1%
	Yes	1392	95.9%
How soon were you able to resume your normal daily activities following the surgery?	1-2 weeks	372	25.6%
	3-4 weeks	396	27.3%
	More than 4 weeks	228	15.7%
	Within a week	456	31.4%
Did you receive any postoperative rehabilitation or physiotherapy?	No	1092	75.2%
	Yes	360	24.8%
How satisfied are you with the overall outcome of your surgery?	Dissatisfied	48	3.3%
	Neutral	132	9.1%
	Satisfied	564	38.8%
	Satisfied	144	9.9%
	Very satisfied	564	38.8%

Table 3 shows that the implementation of ERAS protocols and its association with surgical outcomes are examined. Among participants, 1,152 (79.3%) indicated that ERAS protocols were implemented during their surgical procedure. Fewer individuals experienced surgical site infections 216 (14.9%), and the majority reported healing of surgical incisions within one to two weeks (444, 30.6%). Readmission within 30 days was required by 168 (11.6%), and most participants 1,392 (95.9%) received pain medication within three to seven days. Complications related to anesthesia were reported by 96 (6.6%) of participants.

**Table 3:** ERAS protocol and surgical outcomes ERAS - Enhanced recovery after surgery Surgical site infection categories include infection, bleeding, wound healing issues, and organ dysfunction.

		Count	N %
Were ERAS protocols implemented during your surgical procedure?	No	300	20.7%
	Yes	1152	79.3%
Did you experience any surgical site infections?	No	1236	85.1%
	Yes	216	14.9%
How long did it take for your surgical incision to heal?	1-2 weeks	444	30.6%
	3-4 weeks	408	28.1%
	More than 4 weeks	228	15.7%
	Within a week	372	25.6%
Did you require readmission to the hospital within 30 days of your surgery?	No	1284	88.4%
	Yes	168	11.6%
How many days after surgery did you require pain medication?	1-2 weeks	324	22.3%
	3-7 days	540	37.2%
	Less than 3 days	480	33.1%
	More than 2 weeks	108	7.4%
Did you experience any postoperative nausea or vomiting?	No	1020	70.2%
	Yes	432	29.8%
Did you require blood transfusion during your surgery or hospital stay?	No	1308	90.1%
	Yes	144	9.9%
How would you rate your overall satisfaction with the surgical outcomes?	Dissatisfied	36	2.5%
	Neutral	156	10.7%
	Satisfied	636	43.8%
	Very satisfied	624	43.0%
Did you experience any complications related to anesthesia (e.g., allergic reaction, respiratory issues)?	No	1356	93.4%
	Yes	96	6.6%
How would you rate the overall quality of your recovery process?	Excellent	840	57.9%
	Fair	72	5.0%
	Good	516	35.5%
	Poor	24	1.7%

Table 4 shows that the participants who had ERAS protocols implemented had significantly lower rates of surgical site infections (14.9% vs. 33.3%, p < 0.001) and readmission (11.6% vs. 100%, p < 0.001). ERAS implementation was also associated with reduced days requiring pain medication (p < 0.001) and improved satisfaction with surgical outcomes (p < 0.001). Moreover, participants with ERAS protocols had higher overall satisfaction rates with their recovery process (p < 0.001).

**Table 4:** Bivariate association (chi-square) between the application ERAS protocol and surgical outcomes p-values indicate the statistical significance of associations between ERAS implementation and various surgical outcomes.

		Were ERAS protocols implemented during your surgical procedure?				P-value
		No		Yes		
		Count	Row N %	Count	Row N %	
Did you experience any surgical site infections?	No	228	18.4%	1008	81.6%	<0.001
	Yes	72	33.3%	144	66.7%	
How long did it take for your surgical incision to heal?	1-2 weeks	96	21.6%	348	78.4%	0.222
	3-4 weeks	84	20.6%	324	79.4%	
	More than 4 weeks	36	15.8%	192	84.2%	
	Within a week	84	22.6%	288	77.4%	
Did you require readmission to the hospital within 30 days of your surgery?	No	300	23.4%	984	76.6%	<0.001
	Yes	0	0.0%	168	100.0%	
How many days after surgery did you require pain medication?	1-2 weeks	36	11.1%	288	88.9%	<0.001
	3-7 days	120	22.2%	420	77.8%	
	Less than 3 days	120	25.0%	360	75.0%	
	More than 2 weeks	24	22.2%	84	77.8%	
Did you experience any postoperative nausea or vomiting?	No	204	20.0%	816	80.0%	0.339
	Yes	96	22.2%	336	77.8%	
Did you require blood transfusion during your surgery or hospital stay?	No	276	21.1%	1032	78.9%	0.212
	Yes	24	16.7%	120	83.3%	
How would you rate your overall satisfaction with the surgical outcomes? Did you experience any complications related to anesthesia (e.g., allergic reaction, respiratory issues)?	Dissatisfied	12	33.3%	24	66.7%	<0.001
	Neutral	48	30.8%	108	69.2%	
	Satisfied	132	27.5%	348	72.5%	
	Very satisfied	36	23.1%	120	76.9%	
Did you experience any surgical site infections?	No	228	18.4%	1008	81.6%	<0.001
	Yes	72	33.3%	144	66.7%	
How would you rate the overall quality of your recovery process?	Excellent	144	17.1%	696	82.9%	<0.001
	Fair	36	50.0%	36	50.0%	
	Good	108	20.9%	408	79.1%	
	Poor	12	50.0%	12	50.0%	

Table 5 shows that the participants provided with preoperative instructions for enhanced recovery reported higher satisfaction (1,140, 78.5%). Those who received information about postoperative pain management options 1,008 (69.4%) and were encouraged to actively participate in recovery 900 (62.0%) were more satisfied. Higher satisfaction was also associated with personalized goal-setting 648 (44.6%), regular progress updates 756 (52.1%), and clear self-care instructions 1,320 (90.9%). ERAS participants reported significantly higher satisfaction scores on the scale (p < 0.001).

**Table 5:** ERAS protocol and patient satisfaction Satisfaction questions were measured on a scale from 1 (Dissatisfied) to 5 (Very satisfied).

		Count	N %
Were you provided with specific preoperative instructions to enhance your recovery?	No	312	21.5%
	Yes	1140	78.5%
Did you receive adequate information about postoperative pain management options?	No	444	30.6%
	Yes	1008	69.4%
Were you encouraged to actively participate in your own recovery process?	No	552	38.0%
	Yes	900	62.0%
How satisfied were you with the level of support provided by the healthcare staff during your recovery?	Neutral	228	15.7%
	Satisfied	456	31.4%
	Satisfied	168	11.6%
	Very dissatisfied	12	0.8%
	Very satisfied	588	40.5%
Were you involved in setting personalized goals for your recovery?	No	804	55.4%
	Yes	648	44.6%
Did you receive regular updates on your progress during the recovery process?	No	696	47.9%
	Yes	756	52.1%
How satisfied were you with the overall communication between healthcare providers and yourself during the recovery process?	Dissatisfied	36	2.5%
	Neutral	384	26.4%
	Satisfied	576	39.7%
	Very dissatisfied	12	0.8%
	Very satisfied	444	30.6%
Were you provided with clear instructions regarding self-care and home management after discharge?	No	132	9.1%
	Yes	1320	90.9%

Table 6 shows that the participants who had ERAS protocols implemented (mean satisfaction scale score ± SD: 4.07 ± 0.939) reported significantly higher levels of satisfaction compared to those who did not have ERAS protocols (mean satisfaction scale score ± SD: 2.57 ± 0.719), with a p-value less than 0.001.

**Table 6:** Bivariate association (T-test) between ERAS protocol and postoperative satisfaction Mean satisfaction scores compared between participants with and without ERAS implementation; p-value indicates statistical significance.

	ERAS		P-value
	Yes	No	
Satisfaction scale (Mean ± SD)	4.07 ± 0.939	2.57 ± 0.719	<0.001

## Discussion

This study presents a comprehensive analysis of the influence of ERAS protocols on surgical outcomes, patient satisfaction, and cost-effectiveness within the Saudi Arabian healthcare landscape.

The observations in this cross-sectional study provide valuable insights into the application of ERAS protocols in Saudi Arabian healthcare settings. Among the 1,452 surgical patients analyzed, a notable percentage of participants reported the implementation of ERAS protocols during their surgical procedures, with 1,152 (79.3%) confirming its usage. Importantly, the adoption of ERAS was associated with several significant advantages, such as shorter hospital stays, reduced postoperative complications, decreased rates of readmission, and fewer days requiring pain medication. Additionally, participants in the ERAS group expressed considerably higher satisfaction scores compared to their non-ERAS counterparts. These findings not only align with international and Middle Eastern studies but also extend our understanding of the positive impact of ERAS protocols on postoperative recovery and surgical outcomes [13-18].

The substantial reduction in the length of hospital stays among patients subjected to ERAS protocols holds significant clinical and economic implications. This outcome not only signifies improved patient recovery but also has the potential to reduce healthcare costs, alleviate the burden on healthcare facilities, and enhance bed availability for other patients. These findings resonate with international research, such as the study on ERAS protocols after gastric cancer surgery, which reported a substantial decrease in both length of stay and hospital costs [18].

Another crucial observation is the decline in postoperative complications among ERAS patients. While the reduction in pulmonary infections is in line with prior research, the lack of impact on anastomotic leaks, postoperative complications, and surgical site infections calls for further investigation. These results underscore the complexity of surgical outcomes and the multifaceted nature of complications, which can be influenced by various clinical and patient-specific factors [19].

Equally important is the increase in patient satisfaction scores among those in the ERAS group. This outcome emphasizes that ERAS protocols, with their focus on patient engagement, preoperative education, and comprehensive perioperative care, have the potential to enhance not only physical recovery but also the psychological and emotional well-being of surgical patients. The international literature consistently supports the notion that ERAS protocols lead to higher levels of patient satisfaction [20-22].

The implications of these findings are twofold. First, they provide compelling support for the adoption of ERAS protocols in Saudi Arabia, highlighting their potential to significantly enhance surgical outcomes, reduce hospital costs, and improve patient satisfaction. In a healthcare system that strives to optimize resources and enhance patient experiences, implementing ERAS protocols appears to be a logical step.

Second, these results underline the need for more extensive research on the impact of ERAS on specific surgical procedures and patient groups. While this study provides a broad overview, future research should delve into the nuances of ERAS within different specialties and patient populations. Furthermore, as our study did not directly assess the impact of ERAS on the satisfaction and experience of the surgical team, future investigations should consider the perspectives and experiences of healthcare professionals, as their engagement is vital for the successful implementation of ERAS protocols.

Like all research, this study has limitations. The cross-sectional design restricts our ability to establish causality, making it essential for future studies to explore the long-term effects of ERAS implementation. Additionally, the convenience sampling technique may introduce selection bias, affecting the generalizability of our results. Self-reported data, while valuable, can be subject to response and recall bias. Addressing these limitations through more robust study designs, larger sample sizes, and varied data sources can enhance the validity of future research [13-22].

Given these findings and their associated limitations, several directions for future research are proposed. First, studies should delve into the impact of ERAS protocols on specific surgical procedures and patient populations, considering the unique challenges and benefits within each context. Additionally, research should investigate the experiences and perspectives of healthcare professionals involved in ERAS implementation, offering a comprehensive understanding of the dynamics of this transformative approach.

This study contributes significantly to the growing body of knowledge regarding ERAS protocols within the context of Saudi Arabia. It reinforces the positive impact of these protocols on surgical outcomes, patient satisfaction, and cost-effectiveness. Furthermore, it encourages future research to delve deeper into the potential benefits of ERAS in specific surgical contexts and assess the satisfaction of the surgical team. Ultimately, the adoption of ERAS protocols in Saudi Arabia and other Middle Eastern countries has the potential to significantly improve surgical care and enhance patient experiences. This research serves as a crucial stepping stone in that direction [13-22].

## Conclusions

In our investigation of the impact of implementing ERAS protocols on surgical outcomes and patient satisfaction in the context of Saudi Arabia, we have unearthed compelling evidence that sheds light on the potential benefits of this innovative approach. Our findings reveal a significant reduction in the incidence of surgical site infections, a noteworthy decrease in the duration of hospital stays, and a marked improvement in pain management when ERAS protocols are employed in the perioperative care of patients. Furthermore, the study underscores the substantial enhancement in postoperative satisfaction levels and overall recovery experiences among patients who have undergone surgical procedures with the integration of ERAS protocols. These positive outcomes not only contribute to the overall well-being of patients but also hold the potential to optimize the surgical care process in Saudi Arabia.

It is evident that further research and a deeper focus on the implementation of ERAS protocols are needed in the region, as this approach shows promise in elevating patient outcomes and satisfaction, which are fundamental aspects of modern healthcare. In conclusion, our study not only highlights the importance of integrating ERAS protocols into surgical practices but also signifies their potential to enhance patient care and improve the quality of surgical procedures. As we move forward, we recommend that future studies delve into the intricacies of compliance with ERAS protocols in Saudi Arabian hospitals and focus on long-term assessments of patient quality of life, thereby advancing the knowledge and implementation of ERAS in the field of surgical medicine.
